# Identification and validation of key molecules associated with humoral immune modulation in Parkinson’s disease based on bioinformatics

**DOI:** 10.3389/fimmu.2022.948615

**Published:** 2022-09-15

**Authors:** Na Xing, Ziye Dong, Qiaoli Wu, Pengcheng Kan, Yuan Han, Xiuli Cheng, Biao Zhang

**Affiliations:** ^1^ Clinical College of Neurology, Neurosurgery and Neurorehabilitation, Tianjin Medical University, Tianjin, China; ^2^ Tianjin Key Laboratory of Cerebral Vascular and Neurodegenerative Diseases, Tianjin Neurosurgical Institute, Tianjin Huanhu Hospital, Tianjin, China; ^3^ Department of Clinical Laboratory, Tianjin Huanhu Hospital, Tianjin, China

**Keywords:** ELISA, bioinformatics, biomarker, humoral immune response, gene expression, Parkinson’s disease

## Abstract

**Objective:**

Parkinson’s disease (PD) is the most common neurodegenerative movement disorder and immune-mediated mechanism is considered to be crucial to pathogenesis. Here, we investigated the role of humoral immune regulatory molecules in the pathogenesis of PD.

**Methods:**

Firstly, we performed a series of bioinformatic analyses utilizing the expression profile of the peripheral blood mononuclear cell (PBMC) obtained from the GEO database (GSE100054, GSE49126, and GSE22491) to identify differentially expressed genes related to humoral immune regulatory mechanisms between PD and healthy controls. Subsequently, we verified the results using quantitative polymerase chain reaction (Q-PCR) and enzyme-linked immunosorbent assay (ELISA) in clinical blood specimen. Lastly, receiver operating characteristic (ROC) curve analysis was performed to determine the diagnostic effects of verified molecules.

**Results:**

We obtained 13 genes that were mainly associated with immune-related biological processes in PD using bioinformatic analysis. Then, we selected PPBP, PROS1, and LCN2 for further exploration. Fascinatingly, our experimental results don’t always coincide with the expression profile. PROS1 and LCN2 plasma levels were significantly higher in PD patients compared to controls (p < 0.01 and p < 0.0001). However, the PPBP plasma level and expression in the PBMC of PD patients was significantly decreased compared to controls (p < 0.01 and p < 0.01). We found that PPBP, PROS1, and LCN2 had an area under the curve (AUC) of 0.663 (95%CI: 0.551–0.776), 0.674 (95%CI: 0.569–0.780), and 0.885 (95%CI: 0.814–0.955). Furthermore, in the biological process analysis of gene ontology (GO), the three molecules were all involved in humoral immune response (GO:0006959).

**Conclusions:**

In general, PPBP, PROS1, and LCN2 were identified and validated to be related to PD and PPBP, LCN2 may potentially be biomarkers or therapeutic targets for PD. Our findings also provide some new insights on the humoral immune modulation mechanisms in PD.

## Introduction

With the advancement of medical development, life expectancy has increased greatly; especially as the degree of aging is increasing, the incidence of age-associated neurodegenerative diseases is also enhanced. Parkinson’s disease (PD) is the most common neurodegenerative movement disorder and its onset is usually between 65 and 70 years of age (1). Clinically, resting tremor, bradykinesia, and rigidity are the most important movement symptoms (2). Pathologically, the hallmark of PD is the loss of dopamine neurons in the substantia nigra (SN) and the formation of Lewy bodies containing misfolded α-synuclein (3). To date, the causes of PD still remain unclear.

Neuroimmune inflammation can protect neurons from damage but its toxic effects also exacerbate neuronal disturbance, which is mainly caused by the innate immune cells of the central nervous system (CNS) (microglia and astrocyte) and peripheral invasive immune cells (monocyte, T lymphocyte, B lymphocyte, neutrophil, etc.) regulated by cytokines and chemokines (4, 5). The destruction of the blood brain barrier (BBB) in neurodegenerative disorders allows blood-derived neurotoxic substances, cells, and microbial pathogens entry into the brain, these are associated with inflammatory and immune response, which can initiate a variety of neurodegenerative pathways (6). Inflammatory CNS cells express certain adhesive molecules attracting more peripheral immune cells and antibodies through the BBB into CNS (7). Peripheral interference from systemic inflammation or the gut microbiome can also alter the progression of such injury from microglia (8). With the spread of α-synuclein aggregates into the entire brain region, microglia activation and immune response eventually lead to the degeneration of dopaminergic neurons (9). An exploratory study shows that PD affects the peripheral immune system by inducing changes in the peripheral blood mononuclear cell (PBMC, including lymphocyte and monocyte) compartment (10). The inflammatory progress of the peripheral and central nervous system may be the link between aging, various risk factors, and the development of neurodegenerative disorders (11).

Given that the immune-mediated mechanism is crucial to the pathogenesis of PD, the more thoroughly we explore the role of immune system in PD, the more effective immunotherapy will be found. Microarray or sequencing can detect parallel expression levels for thousands of genes in tissues or body fluids, enabling the rapid scanning of multiple genes and screening of candidate biomarkers. Recently, hundreds of gene expression datasets about PD have been uploaded to the Gene Expression Omnibus database (GEO). However, most studies are based on a single cohort analysis; thus, the results sometimes are inconsistent and the identification of molecular pathways involved in PD still lacks reliability. Different reports indicate that PD can also affect PBMCs of the peripheral immune system (12–14). Therefore, we downloaded the datasets of PBMC in human with PD from GEO, screened out the differentially expressed genes (DEGs), and performed a series of functional analysis. Some molecules are enriched in several immune response pathways, which are primarily associated with humoral immune modulation. These molecules may potentially be used as diagnostic biomarkers and therapeutic targets for PD.

## Materials and methods

**Figure 1:**
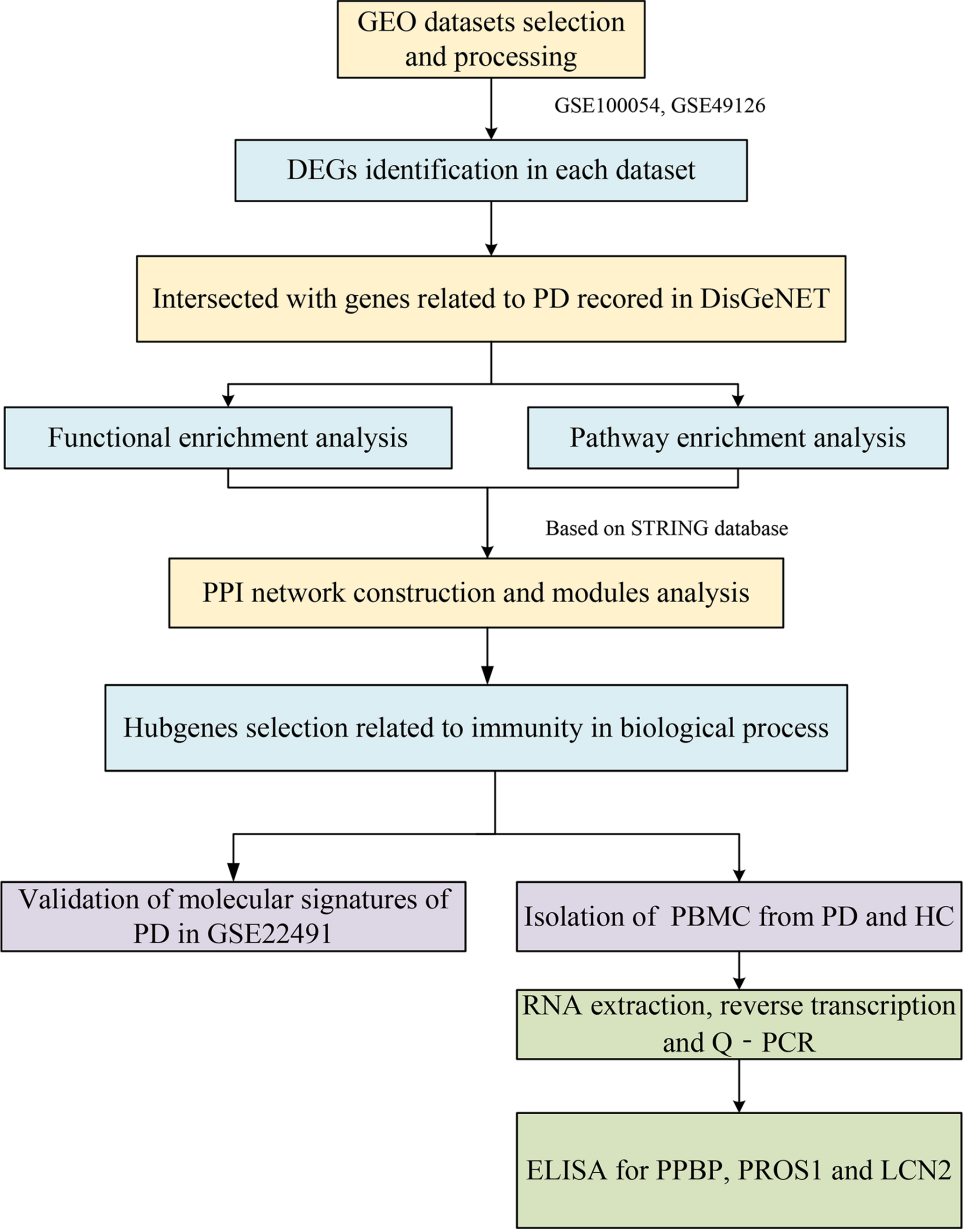
The workflow of this study.

The workflow of this study is shown in **Figure 1**.

### Data collection and preprocessing

Gene expression data GSE100054 (15) (10 PD and 9 HC) and GSE49126 (16) (30 PD and 20 HC) were downloaded from the Gene Expression Omnibus Database (GEO) (https://www.ncbi.nlm.nih.gov/geo/). GSE22491 (17) (10 PD and 8 HC) was used as a validation data set. We used the GEOquery package in R (version 4.0.4, 2021-02-15) (18) to download the expression matrix and platform information then performed principal component analysis (PCA) to observe the distribution between groups. The expression matrix was processed according to the following screening criteria: (1) removing the empty probe that did not match any gene, (2) deleting the probe corresponding to multiple genes, and (3) if a gene corresponded to more than one probe, the probe with the highest expression represented the expression level of this gene.

### Differentially expressed gene identification

A linear model was employed to assess the differential expression between PD patients and healthy controls using an R package called “limma” (19). Benjamini and Hochberg’s method (BH) was used to control the false discovery rate across all the genes. The DEGs were screened at p < 0.05 and |log2FC| > 1 in the GSE100054, and at p < 0.05 and |log2FC| > 0.5 in the GSE49126. All DEGs intersected with the genes associated with Parkinson’s disease recorded in the disease database DisGeNET (https://www.disgenet.org/) (20) to obtain DEGs of interest.

### PPI network construction and module analysis

The protein–protein interaction network (PPI) was constructed using the Search Tool for the Retrieval of Interacting Genes (STRING; https://cn.string-db.org/). The STRING database is an online search tool containing information on known and predicted protein interactions. The protein interaction information comes from these aspects including textmining, experiments, databases, co-expression, neighborhood gene fusion, and co-occurrence. We uploaded the DEGs of interest to the STRING database to establish a PPI network. In this study, protein interactions with a connection score >0.4 were considered statistically significant and remained in the PPI network. After downloading PPI analysis results, we input it into the cytoscape software (version 3.8.2) for visualization and module analysis using the plug-in molecular complex detection (MCODE).

### KEGG and GO enrichment analysis of DEGs and significant module

An enrichment analysis of the Gene Ontology (GO) (21) and Kyoto Encyclopedia of Genes and Genomes (KEGG) pathways (22) for DEGs of interest and significant module was performed using the clusterProfiler package in R (23). We performed GO term enrichment analysis under the following three sub-ontologies: biological process (BP), molecular function (MF), and cellular component (CC). The GO classifications were evaluated using Fisher’s exact test to obtain p-values, which were filtered at a cut-off value of 0.05. Using the key term “immune”, we identified 39 pathways involved in immune-related biological process and obtained 13 molecules in total: CCR2, CD44, TLR2, TLR4, IL4, IL-1B, IL-33, TGFB2, PPBP (CXCL7/NAP-2), PROS1, CLU, LCN2 (NGAL), and NLRP3.

### Validation of molecular signatures in PD

In the data set GSE22491, we compared the expression level of 13 molecules in PD patients and healthy controls. The expression of other genes was significantly different between the two groups except for IL-1B, TGFB2, CLU, IL33, and NLRP3. Considering the overall feasibility and innovation of subsequent experiments, the three molecules of PPBP, PROS1, and LCN2 were selected for further verification. The study was approved by the Ethics Committee of Tianjin Huanhu Hospital, and all participants provided informed consent. We enrolled 69 PD patients from three specialists in neurodegenerative diseases according to the UK bank criteria based upon clinical and neuropsychological examination, and 36 age- and sex-matched healthy controls. Family history was determined by interviewing or reviewing medical records without genetic testing to minimize the inherited form. Any other neurological and mental disorder, cardiovascular or cerebrovascular event, acute or chronic inflammation, or cancer was excluded. Participants who had received anti-inflammatory treatment in the past 2 months were not included. Healthy controls were included without any history of neurological or psychiatric disorders. Levodopa or dopamine agonists may interfere with different central neurotransmitter pathways and affect gene expression profiles; in addition, some works have reported that dopamine had effects on immune cells (24, 25). Therefore, subjects had not taken any drugs acting on the CNS in the past 6 months. Demographic data and clinical characteristics of participants in this study are summarized in **Table 1**.

**Table 1:** The demographic data and clinical characteristics of participants in this study.

Variable	PD (n = 69)	HC (n = 36)	Group comparison
Age, years	65.96 ± 8.961	68.75 ± 9.82	0.145
Male/female ratio	39/30	19/17	0.714
H&Y stage, off (1|1.5|2|2.5|3|4|5|)	5|7|10|7|27|13|0		
MDS-UPDRS III “off” (0-132)	49.09 ± 18.76		
Disease duration, years	7.27 ± 3.98		
WBC, 10^9/L	6.09 ± 2.06	5.72 ± 1.44	0.448
Neutrophils (%)	62.40 ± 10.02	58.51 ± 8.10	0.080
Lymphocyte (%)	28.99 ± 8.85	32.62 ± 7.66	0.040
Monocytes (%)	6.05 ± 1.43	6.14 ± 1.42	0.765
N/L	2.83 ± 2.91	1.98 ± 0.86	0.060
M/L	0.24 ± 1.46	0.20 ± 0.08	0.098

Values are means ± SD unless otherwise stated. PD, Parkinson’s disease; HC, healthy controls. WBC, leukocyte; N/L, neutrophil/lymphocyte; M/L, monocyte/lymphocyte.

### PBMC isolation, RNA extraction, and reverse transcription

The 2 mL fasting sample of venous whole blood was drawn from each participant into the EDTA anticoagulant tube on the next day of hospitalization. The whole blood was centrifuged at 600×*g* for 5 min at room temperature and the plasma on top was centrifuged at 1,000×*g* for 15 min once again, then stored at -80°C until further analysis. The bottom cell layer was diluted with an equal amount of PBS (Solarbio, Beijing, China). PBMCs were isolated using Ficoll‐Hypaque (TBD, Tianjin, China) with density gradient centrifugation according to the protocol. Firstly, centrifugation was at 400×*g* for 20 min on a horizontal rotor centrifuge; thus, we absorbed the second layer from top to bottom, which was resuspended in PBS and centrifuged at 250×*g* for 10 min in triplicate. Next, RNA was extracted from PBMCs with a TRIzol reagent (Invitrogen, USA) according to the manufacturer’s instructions; the RNA concentrations were then evaluated using the Nanodrop spectrophotometer. In addition, we also extracted RNA from whole blood; a blood (liquid sample) total RNA rapid extraction kit (spin column type) (BioTeke, Beijing, China) was applied (**Supplementary Methods 1**). cDNA synthesis was conducted using the FastKing gDNA Dispelling RT SuperMix (Tiangen, Beijing, China) and the reaction was incubated at 42°C for 15 min (cDNA synthesis) and terminated by heating at 95°C for 3 min. Finally, cDNA was stored at -80°C.

### Quantitative real-time polymerase chain reaction

Quantitative real-time polymerase chain reaction (Q-PCR) was performed by SuperReal PreMix Plus (SYBR Green) (Tiangen, Beijing, China) on the Roche LightCycler 480, and the primer sequences are shown in **Table 2**. The thermal cycling program was as follows: 95°C 15 min, 40 cycles of 95°C for 10 s, and 60°C for 30 s. A housekeeping gene (GAPDH) was used as an endogenous control for normalization. The expression of target genes was normalized to GAPDH using the 2−ΔΔCT method.

**Table 2:** The primer sequences used for Q-PCR.

Gene	Forward primer sequence (5′→3′)	Reverse primer sequence (5′→3′)
GAPDH	TCAAGGCTGAGAACGGGAAG	CGCCCCACTTGATTTTGGAG
PROS1	TTGCACTTGTAAACCAGGTTGG	CAGGAACAGTGGTAACTTCCAG
LCN2	GGTATGTGGTAGGCCTGGCA	AACAGGACGGAGGTGACATTGT
CXCL7	TTGTAGGCAGCAACTCACCC	TGCAAGGCATGAAGTGGTCT

### Enzyme-linked immunosorbent assay

The concentrations of PPBP, PROS1, and LCN2 in the plasma of PD and HC were measured using commercially available enzyme-linked immunosorbent assay kits (CUSABIO, Wuhan, China) in accordance with manufacturer’s instructions, which were detected at 450 nm with a microplate reader and calculated by comparing the OD value of the samples to a standard curve. We made a standard curve using the “Curve Expert” software, which was downloaded from CUSABIO’s web. The concentration read from the standard curve must be multiplied by the dilution factor.

The plasma concentration of LCN2 was determined by human NGAL ELISA kit (CSB-E09408h; CUSABIO, Wuhan, China). The measurement range of this kit was 0.0156–1.0 ng/mL; the coefficient of variation for intra-assay and inter-assay was <8% and <10%, respectively. The minimum detection limit was 0.0039 ng/mL.

The plasma concentration of PROS1 was determined using a human PROS1 ELISA kit (CSB-E09903h; CUSABIO, Wuhan, China). The measurement range of this kit was 3.12–200.0 ng/mL; the coefficient of variation for intra-assay and inter-assay was <8% and <10%, respectively. The minimum detection limit was 0.78 ng/mL.

The plasma concentration of PPBP was determined using a human NAP-2 ELISA kit (CSB-E04562h; CUSABIO, Wuhan, China). The measurement range of this kit was 0.078–5.0 ng/mL; the coefficient of variation of intra-assay and inter-assay was <8% and <10%, respectively. The minimum detection limit was 0.0195 ng/mL.

### Statistical analysis

Statistical analysis was performed using the GraphPad Prism 8 software and SPSS 26.0 software. For all analysis, statistical significance was taken as a two-sided p-value <0.05. Data normality was first evaluated using the Shapiro–Wilk test; if normality assumption was satisfied, data were presented as mean ± standard deviation (SD), and the significant differences between groups were further analyzed using the Student’s *t*-test. If not, median (Q1-Q3) described the data distribution characteristics and the Mann–Whitney *U* test was applied (Wilcoxon rank sum test). chi-Square tests compared sex ratios between PD patients and healthy controls. Receiver operating characteristic (ROC) curves were generated to evaluate their sensitivities and specificities in distinguishing PD from healthy controls.

## Results

### Identification of differentially expressed genes

**Figure 2:**
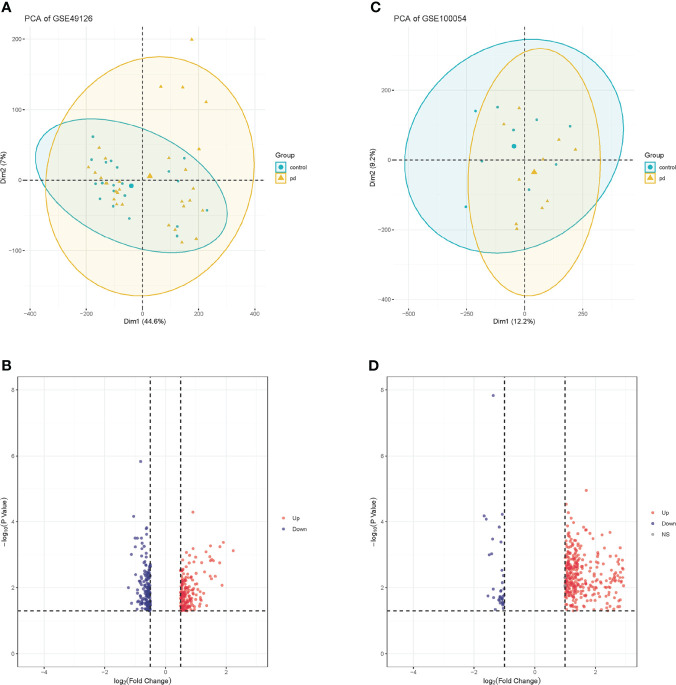
PCA plots of gene chips and volcano maps of different expression genes. **(A)** PCA analysis plot of GSE100054 gene chip; **(B)** Volcano map of GSE100054 gene chip; **(C)** PCA analysis plot of GSE49126 gene chip; **(D)** Volcano map of GSE49126 gene chip. Control represents healthy controls, pd represents Parkinson’s disease. Up represents upregulated genes, and down represents downregulated genes.

**Figure 3:**
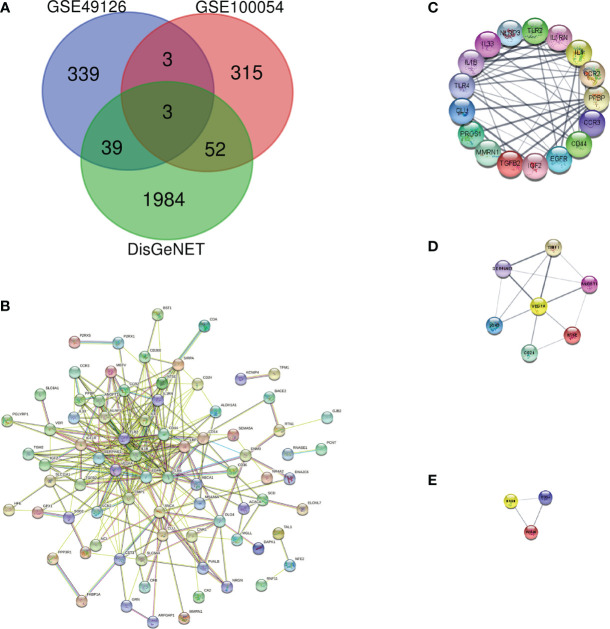
Protein–protein interaction network and key protein expression module. **(A)** Venn diagram of DEGs in GSE100054, GSE49126 gene chips and genes from DisGeNET; **(B)** Protein–protein interaction network of DEGs; **(C)** Key protein expression module 1; **(D)** Key protein expression module 2; **(E)** Key protein expression module 3. The nodes represent genes, and the edges represent links between genes.

We downloaded the microarray expression data set from the GEO database. After a differential expression analysis according to the above selection criteria, we obtained 373 DEGs including 337 upregulated genes and 36 downregulated genes in GSE100054, and 384 DEGs including 200 upregulated genes and 184 downregulated genes in GSE49126. The principal component analysis (PCA) was performed and shown by the scatter plot, in which each point represented a sample (**Figures 2A, C**) and the screened genes with significant difference between groups were displayed by different colors in the volcano map (**Figures 2B, D**). GSE100054 and GSE49126 were used to identify differently expressed genes (DEGs), then intersected with the recorded PD-related genes by DisGeNET database, respectively (**Figure 3A**). A total of 94 target genes are shown in **Supplementary Table 1**.

### PPI network analysis

To describe the interactions between proteins encoded by differentially expressed genes, we constructed a PPI network from the STRING database (**Figure 3B**). The network consisted of 92 nodes and 293 edges; nodes represented DEGs of interest and edges represented interactions between genes. The PPI enrichment p-value was <1.0e-16, then the PPI network was imported into cytoscape for visualization, and MCODE was used to perform a cluster correlation analysis with default Node Score Cutoff as 0.2, K-core as 2, and Max.Depth as 100. At last, three clusters with high scores were obtained, namely, Cluster 1 (17 nodes, score 8.7, **Figure 3C**), Cluster 2 (7 nodes, score 4.3, **Figure 3D**), and Cluster 3 (3 nodes, score 3.0, **Figure 3E**). A total of 27 Hubgenes are displayed in **Table 3**.

**Table 3:** The 27 Hubgenes of three clusters.

Symbol	ENTREZID	Gene Name
IL1RN	3557	Interleukin 1 receptor antagonist (IL1RN)
PROS1	5627	Protein S (alpha) (PROS1)
SERPINE1	5054	Serpin family E member 1 (SERPINE1)
CLU	1191	Clusterin (CLU)
EGFR	1956	Epidermal growth factor receptor (EGFR)
NRGN	4900	Neurogranin (NRGN)
NT5E	4907	5′-nucleotidase ecto (NT5E)
NLRP3	114548	NLR family pyrin domain containing 3 (NLRP3)
TIMP1	7076	TIMP metallopeptidase inhibitor 1 (TIMP1)
CCR3	1232	C-C motif chemokine receptor 3 (CCR3)
CCR2	729230	C-C motif chemokine receptor 2 (CCR2)
IL33	90865	Interleukin 33 (IL33)
TGFB2	7042	Transforming growth factor beta 2 (TGFB2)
ANGPT1	284	Angiopoietin 1 (ANGPT1)
IGF2	3481	Insulin like growth factor 2 (IGF2)
PPBP	5473	Pro-platelet basic protein (PPBP)
VEGFA	7422	Vascular endothelial growth factor A (VEGFA)
IL4	3565	Interleukin 4 (IL4)
MMRN1	22915	Multimerin 1 (MMRN1)
DLG4	1742	Discs large MAGUK scaffold protein 4 (DLG4)
IL1B	3553	Interleukin 1 beta (IL1B)
LCN2	3934	Lipocalin 2 (LCN2)
CD24	100133941	CD24 molecule (CD24)
TLR4	7099	Toll like receptor 4 (TLR4)
CD44	960	CD44 molecule (Indian blood group) (CD44)
PVALB	5816	Parvalbumin (PVALB)
TLR2	7097	Toll like receptor 2 (TLR2)

### Functional enrichment of DEGs and significant module

Enrichment analysis for the DEGs of interest (94 genes) and Hubgenes (27 genes) was carried out by the clusterProfiler package based on *Homo sapien* background to obtain the enrichment information. Through GO classification and enrichment analysis, such as biological process, cell component, and molecular function, functional annotation terms related to PD were revealed and their biological significance was explored. In order to uncover the biological pathways associated with DEGs of interest and Hubgenes, important signaling pathways were obtained using the KEGG database.

**Figure 4:**
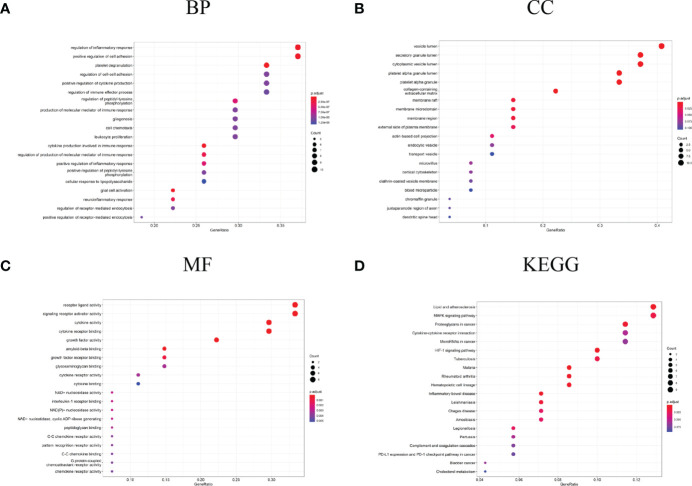
GO and KEGG analysis results of DEGs. **(A)** GO biological process enrichment results; **(B)** GO cell component enrichment results; **(C)** GO molecular function enrichment results; **(D)** KEGG pathway enrichment results. GO: gene ontology. KEGG: Kyoto Encyclopedia of Genes and Genomes.

The biological process in which DEGs were enriched mainly included the regulation of inflammatory response, platelet degranulation, regulation of the immune effector process, production of the molecular mediator of immune response, leukocyte proliferation, cytokine production involved in immune response, neuroinflammatory response, etc. (**Figure 4A**). In terms of “cell component”, DEGs were mainly enriched in the vesicle lumen, platelet alpha granule lumen, external side of plasma membrane, transport vesicle, and other regions (**Figure 4B**); as for the “molecular function”, DEGs were mainly related to cytokine activity, receptor ligand activity, NAD+ nucleosidase activity, interleukin-1 receptor binding, etc. (**Figure 4C**). In the enrichment analysis of KEGG, DEGs mainly are involved in lipid and atherosclerosis, MAPK signaling pathway, cytokine-cytokine receptor interaction, HIF-1 signaling pathway, inflammatory bowel disease and cholesterol metabolism, etc. (**Figure 4D**).

**Figure 5:**
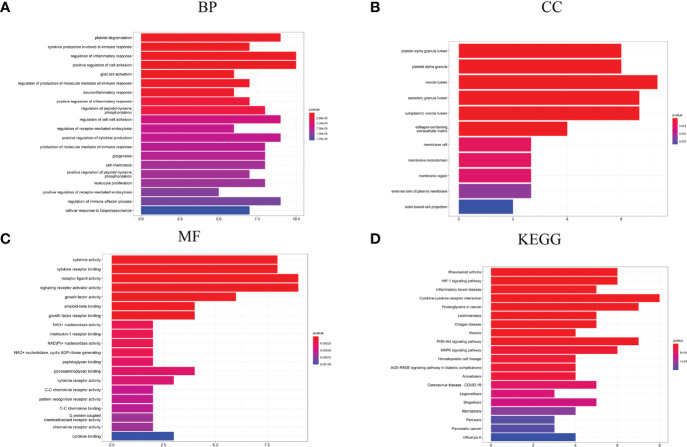
GO and KEGG analysis results of Hubgenes. **(A)** GO biological process enrichment results; **(B)** GO cell component enrichment results; **(C)** GO molecular function enrichment results; **(D)** KEGG pathway enrichment results. GO: gene ontology. KEGG: Kyoto Encyclopedia of Genes and Genomes.

The biological process in which Hubgenes were enriched mainly included cytokine production involved in immune response, regulation of inflammatory response, platelet degranulation, glial cell activation, positive regulation of cytokine production, regulation of the immune effector process, etc. (**Figure 5A**). In terms of “cell component”, Hubgenes were mainly enriched in the vesicle lumen, secretory granule lumen, membrane region, and other regions (**Figure 5B**). As for the “molecular function”, it indicated that Hubgenes were mainly related to cytokine activity, cytokine receptor binding, amyloid-beta binding, C-C chemokine receptor activity, etc. (**Figure 5C**). In the enrichment analysis of KEGG, Hubgenes were mainly involved in rheumatoid arthritis, cytokine-cytokine receptor interaction, proteoglycans in cancer, PI3K-Akt signaling pathway, necroptosis, AGE-RAGE signaling pathway in diabetic complications, etc. (**Figure 5D**).

### Identification and validation of molecular signatures in PD

**Figure 6:**
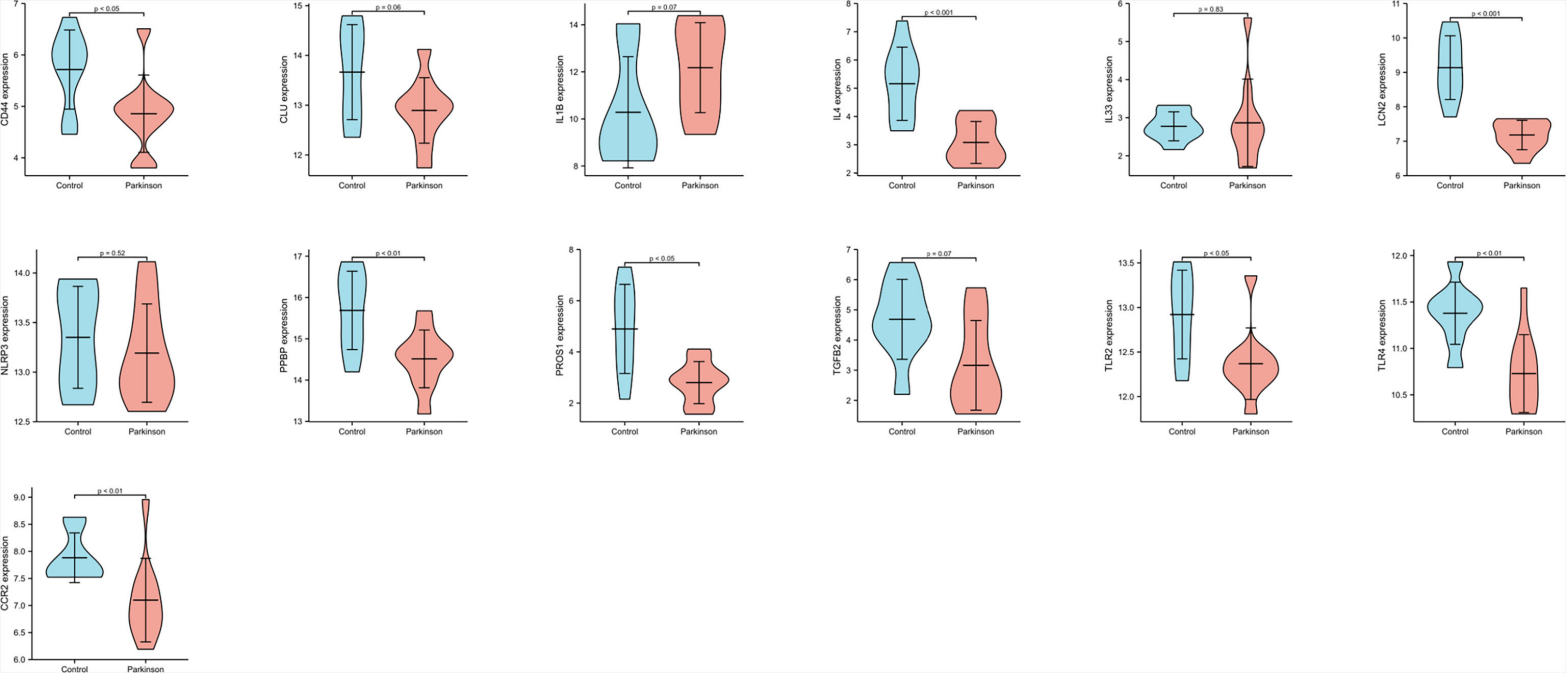
The expression levels of 13 genes in GSE22491. p < 0.05 was considered to be statistically different.

**Figure 7:**
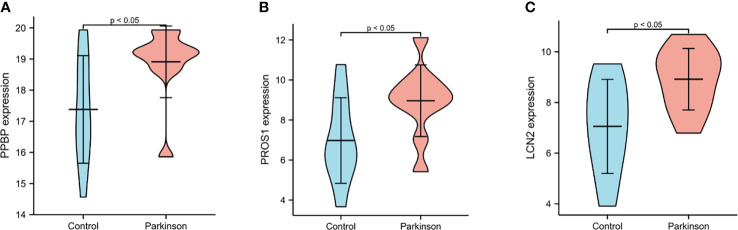
The expression levels of PPBP, PROS1 and LCN2 from PD patients and healthy controls in GSE100054. **(A–C)** The expression of PPBP, PROS1 and LCN2 in GSE100054. p < 0.05 was considered to be statistically different.

We performed the biological process analysis of gene ontology (GO) based on 27 Hubgenes (“immune” as the key word, p < 0.05 as a criterion), screened 39 immune-related pathways (**Supplementary Table 2**), and selected 13 target molecules from the above Hubgenes, such as CCR2, CD 44, TLR2, TLR4, IL4, IL-1B, IL-33, TGFB2, PPBP, PROS1, CLU, LCN2, and NLRP3. GSE22491 was selected as a validation data set to compare the expression levels of these molecules in PD and HC. The expression of IL-1B, TGFB2, CLU, IL33, and NLRP3 had no significant differences between the two groups; other genes were deemed to be significantly different (p < 0.05) (**Figure 6**). We selected three molecules: PPBP, LCN2, and PROS1 for the following experiment based on previous studies and objective conditions of experimental implementation. These three molecules were all decreased in PD compared to HC (**Figure 6**); however, the expression levels of PPBP, PROS1 and LCN2 in PD were higher than HC in GSE100054 (**Figure 7A-C**). Of the immune-related biological processes, PPBP and LCN2 were mainly involved in humoral immune response and neutrophil activation involved in immune response; however, in addition to humoral immune response, PROS1 also participated in the regulation of humoral immune response and the immune effector process (**Table 4**).

**Table 4:** The immune-related pathways in the term of biological process.

ID	Description	GeneRatio	BgRatio	p-value	gene ID
GO:0002283	Neutrophil activation involved in immune response	4/27	490/18866	4.91E-03	PPBP/LCN2/CD44/TLR2
GO:0002697	Regulation of immune effector process	9/27	470/18866	1.08E-08	PROS1/CLU/NLRP3/CCR2/IL33/TGFB2/IL4/IL1B/TLR4
GO:0002920	Regulation of humoral immune response	3/27	134/18866	9.05E-04	PROS1/CLU/IL1B
GO:0006959	Humoral immune response	6/27	377/18866	1.27E-05	PROS1/CLU/CCR2/PPBP/IL1B/LCN2

### Validation of selected differential molecules on transcription level

Sixty-nine patients with PD (39 males and 30 females) and 36 healthy controls (19 males and 17 females) were recruited. From these participants, firstly, we selected some for mRNA expression level analysis.

**Figure 8:**
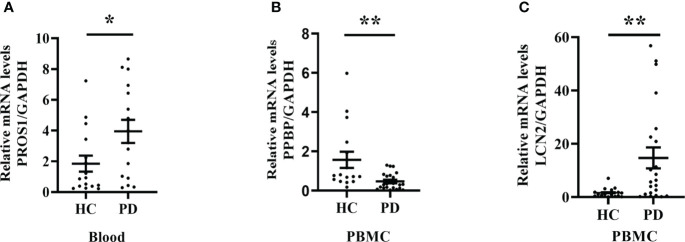
PROS1, PPBP, and LCN2 expression from PD patients and healthy controls. **(A)** The PROS1 expression in whole blood from PD (n = 16) was higher than HC (n = 16); **(B)** The PPBP expression in PBMC from PD (n = 22) was lower than HC (n = 16); **(C)** The LCN2 expression in PBMC from PD (n = 22) was higher than HC (n = 16). HC represents healthy controls, PD represents Parkinson’s disease. * p < 0.05, ** p < 0.01.

We observed that LCN2 expression was significantly increased in PBMC from PD patients compared to the healthy controls; however, the mRNA level of PPBP was decreased (**Figures 8B, C**). The PROS1 expression level was rather low, therefore being unable to assess. In addition, we also extracted RNA from whole blood and the PROS1 expression was significantly increased in PD (**Figure 8A**), while LCN2 and PPBP had no significant differences between groups (**Supplementary Figure 1**).

### Validation of selected differential molecules on translation level

**Figure 9:**
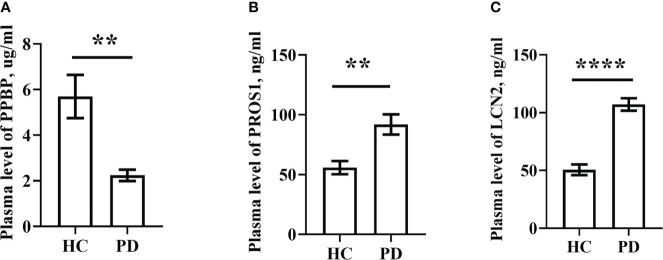
Plasma levels of PPBP, PROS1 and LCN2 in PD patients (n=69) and healthy controls (n=36). **(A)** Comparison of the expression levels of plasma PPBP; **(B)** Comparison of the expression levels of plasma PROS1; **(C)** Comparison of the expression levels of plasma LCN2. HC represents healthy controls, PD represents Parkinson’s disease. ** P < 0.01, **** P < 0.0001.

**Figure 10:**
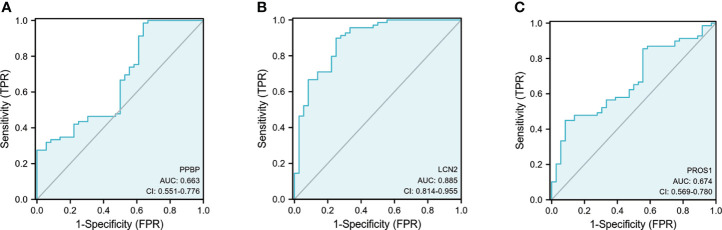
Receiver operating characteristic (ROC) curves of PPBP, ROS1, and LCN2. **(A–C)** The ROC of PPBP, ROS1, and LCN2. sensitivity (true-positive rate) and 1-specificity (false-positive rate); AUC: area under curve; CI: 95% confidence interval.

For ELISA, the plasma levels of the above three molecules were consistent with the mRNA expression. Our findings revealed that PPBP levels in the peripheral blood samples of patients with PD and healthy controls were 1.770 µg/mL (range 0.019–9.839 µg/mL) and 2.269 µg/mL (range 0.692–16.83 µg/mL) respectively (**Figure 9A**). In comparison with the healthy controls, the assay showed that the plasma concentrations of PROS1 and LCN2 both were enhanced in PD (PD: PROS1, 77.52 ng/mL, range 3.727–387.4 ng/mL; LCN2, 98.83 ng/mL, range 41.64–224.6 ng/mL) (HC: PROS1, 55.49 ng/mL, range 2.019–151.6 ng/mL; LCN2, 44.94 ng/mL, range from 14.25–143.2 ng/mL) (**Figures 9B, C**). Receiver operating characteristic (ROC) curves were applied to determine the diagnostic effect of the above three molecules. We found that PPBP, PROS1, and LCN2 with an area under the curve (AUC) of 0.663 (95% CI: 0.551–0.776), 0.674 (95% CI: 0.569–0.780), and 0.885 (95% CI: 0.814–0.955) (**Figures 10A–C**) (**Table 5**).

**Table 5:** Receiver operating characteristic (ROC) of PPBP, PROS1, and LCN2 for prediction of Parkinson’s disease and healthy controls.

Indicator	Sensitivity(95% CI)	Specificity(95% CI)	AUC(95% CI)	Cut-off	Positive LR	Negative LR
PPBP	0.986 (0.911–0.999)	0.361 (0.213–0.537)	0.663 (0.551–0.776)	9.006 µg/mL	1.543 (1.205–1.975)	0.040 (0.005–0.309)
PROS1	0.449 (0.331–0.573)	0.917 (0.764–0.978)	0.674 (0.569–0.780)	85.771 µg/mL	5.391 (1.769–16.432)	0.601 (0.483–0.747)
LCN2	0.899 (0.796–0.955)	0.750 (0.575–0.873)	0.885 (0.814–0.955)	62.726 ng/mL	3.594 (2.030–6.364)	0.135 (0.065–0.278)

## Discussion

The study analyzed the DEGs of interest and Hubgenes in PBMC from PD patients and healthy controls. In terms of “biological process”, DEGs and Hubgenes were mainly enriched in the regulation of inflammatory response, positive regulation of cytokine production, regulation of immune effector process and so on. The PPI network identified 13 genes of interest, and key module analysis showed that these genes were mainly involved in the immune response. We then selected PPBP, PROS1, and LCN2 to validate their expression levels in PBMC and whole blood by Q-PCR and in plasma by ELISA. The plasma level of PPBP, PROS1, and LCN2 were consistent with the mRNA expression. The three molecules above were all reduced in PD in GSE22491 (**Figure 6**) but increased in GSE100054 (**Figure 7**), but we found that PPBP was decreased in PD, and PROS1 and LCN2 were increased compared to healthy controls. Nonetheless, all the evidence suggests that PROS1, PPBP, and LCN2 are known to be well detectable *in vivo* in blood and thus represents a potentially valuable source of biomarkers for Parkinson’s diseases.

GO and KEGG analysis indicated that PROS1, PPBP, and LCN2 were mainly involved in immune-related biological processes associated with humoral immune response. The activation of the innate immune system components, especially endogenous microglia and infiltrating monocytes/macrophages from the periphery, serve a prominent role in the pathogenesis of PD (8, 26, 27). Previous studies also supported the involvement of the adaptive immune system in the pathophysiology and progression of PD (28, 29). In addition, during the inflammatory progress of CNS, peripheral myeloid cells (such as monocytes) can be recruited into the brain through disturbed BBB and further participate in the inflammatory processes (27). Peripheral immune activation is enhanced in PD patients, and dynamic changes in the percentage of CD4+ T cells and IgG level suggest that peripheral immunity plays a positive role in initiation or exacerbation of neurodegeneration (30). The autoantibodies against α-synuclein were related to disease status in the serum and cerebrospinal fluid of PD patients (31); furthermore, a study confirmed that the repertoire of high-affinity anti-α-synuclein autoantibodies was significantly reduced in prodromal phases of PD (32), which indicates that impaired reactivity towards α-synuclein occurs prior to disease onset and humoral immune response is involved in the pathophysiology of neurodegeneration. A recent study also reported that peripheral humoral immune response (particularly serum C3 and IgG) may be correlated with the non-motor symptoms (NMSs) of PD (33).

Chemokines are a class of small secreted proteins that have certain chemotactic effects on different target cells *via* binding to G protein-coupled receptors. CXC chemokines belong to the soluble subfamily of cytokines with low molecular weight and conserved N-terminal CXC motif (34). To date, 17 CXC chemokines have been identified (34), and many studies have confirmed that chemokines play an important role in the immune and nervous system, which can induce peripheral immune cells to reach inflammatory sites, triggering immune responses that lead to the destruction of BBB (35, 36). Activated microglia can upregulate MHC class II molecules and produce cytokines or its corresponding receptors during inflammation (37). Given their effects in cell migration and immune coordination, chemokines may be major candidates for connecting peripheral and central inflammation and coordinating neuroinflammation (38). PPBP, also known as NAP-2/CXCL7, belongs to the CXCL subgroup, which is released by activated platelets and associated with the initiation and development of various tumors; therefore, serum levels of PPBP can be regarded as an auxiliary diagnostic marker of colorectal cancer (CRC) and obstructive colorectal cancer (OCRC) (39, 40). A meta-analysis including 11 studies involving 771 participants showed that blood PPBP levels were significantly higher in depressed patients compared to non-depressed controls (38). Krishna et al. reported that the binding of chemokines (CXCL1, CXCL2, CXCL3, CXCL5, CXCL6, CXCL7, and CXCL8) to corresponding receptors (CXCR1 and CXCR2) triggered the signaling pathway of G-protein or β-arrestin coupling, which played an important non-overlapping part in the trafficking and activation of neutrophils (41). In addition, in the mouse model, overexpressing human full-length α-synuclein could induce the infiltration of CCR2 positive monocytes into the substantia nigra (14). Conversely, loss of CCR2 prevented α-synuclein-induced monocyte infiltration and subsequent dopaminergic neuron death (14). A Korean study showed that acupuncture at GB34 and LR3 might inhibit the degeneration of dopamine neurons in the SN of MPTP-induced Parkinson’s disease mouse model, and the affymetrix gene chip test showed that PPBP was upregulated in MPTP group compared with control; however, compared with the MPTP group without acupuncture, PPBP was downregulated after acupuncture, indicating that PPBP may be involved in the pathogenesis and regulation of PD (42). However, in this study, we observed that blood PPBP level was reduced in PD, which contradicted with previous reports. This inconsistent result may be related to individual differences, methods of sample collection, choices of assays, disease severity, and other confounding variables.

TAM receptors (Tyro3, Axl, and Mertk) are a subgroup of the tyrosine kinase receptor family. TAMs may play a role in the pathogenesis of PD by affecting the activation and phagocytosis of microglia and regulating the deposition of α-synuclein (43). Two ligands of TAM receptors (PROS1 and Gas6) are vitamin K-dependent proteins, and high level of PROS1 can be measured in plasma, which not only plays an independent anticoagulant activity and acts as cofactors in the decomposition of coagulation factors (44) but also executes the phagocytosis of apoptotic cells (45). In the CNS, PROS1 is expressed at a low level, mainly in the locus coeruleus and choroid plexus, but at high levels in human neurons (45), which phosphorylates Mertk and Tyro3 but not Axl (43). Activated human T cells secrete PROS1 that limited dendritic cell activation through TAM receptors and reduced inflammation (46). Another work assessed the plasma concentrations of total and free PROS1 in 65 patients with multiple sclerosis (MS) and 15 controls; total PROS1 in MS was reduced compared with control, while plasma-free PROS1 was extremely lower in patients with higher disease severity, suggesting that the PROS1 level may be a potential biomarker of disease progression (47). In comparison with wild-type mice, the expression of PROS1 was increased in the hippocampus of 5XFAD mice (AD mouse model), and the level of serum PROS1 in 5XFAD mice increased with disease progression. More significantly, PROS1 was also observed enhanced in the serum of AD patients and closely related to human AD neuroimaging markers (48). We also observed that the plasma PROS1 level displayed a significant difference between PD and HC. Therefore, PROS1 can be used as a new plasma biomarker for PD.

Lipocalin-2 (LCN2), also known as neutrophil gelatinase-associated lipocalin (NGAL), 24p3 or ferriportin, is a member of the lipocalin family of transport proteins and acts as a multifunctional mediator of metabolic inflammatory process (11). As a group of highly heterogeneous extracellular proteins, LCN2 induces the activation of microglia and astrocytes through its neurotoxic properties and causes neuronal cell death under neuroinflammation (49). It was reported that LCN2 increased in the SN and serum of PD patients, which was positively correlated with α-synuclein and Aβ40 in CSF (50, 51). The mRNA and protein expression levels of LCN2 increased in the substantia nigra and striatum in two neurotoxin mouse PD models (MPTP and 6-OHDA), and LCN2 promoted neuro-inflammation and neuronal death, leading to the disruption of the dopamine pathway in the SN which caused motor behavior disorder (51). Our results indicated that plasma LCN2 increased in PD; however, another study pointed out that serum LCN2 levels in PD patients did not significantly increase compared to controls, and there was no significant difference in concentration between early and late, cognitively impaired, cognitively normal PD and HC (52). Neuro-inflammation, iron dysregulation, white matter lesions, and BBB destruction are common neuropathological processes in many CNS diseases, and accumulating evidence suggests that LCN2 is associated with iron homeostasis dysregulation, metabolic alteration, and insulin resistance (49). LCN2 has been proposed as a biomarker for preclinical Alzheimer’s disease and vascular dementia (53, 54); its usage as a potential biomarker for PD still needs to be further explored.

This study has some limitations. Firstly, these results were obtained in a small group of participants. The inconsistent results may be related to methods of sample collection, choices of assays, disease severity, drug treatment, and other confounding variables. Therefore, the above findings in this study are still preliminary. Due to the inconvenience and difficulty of conducting long follow-up studies, more detailed clinical information and scale assessment data cannot be obtained. Comprehensive clinical information is conducive to the objective evaluation of research results, and we will strengthen the acquisition of clinical data of patients to enable subgroup analysis in the future.

## Conclusion

In this study, we used the gene expression dataset to identify DEGs and found 13 genes of interest by constructing a PPI network and identifying key modules. Based on the biological process analysis of Gene ontology (GO), PPBP, PROS1, and LCN2 have been involved in humoral immune response (GO:0006959). Although we have proved that PPBP, PROS1, and LCN2 were statistically different in PD patients and healthy controls using Q-PCR and ELISA, further investigations are needed to yield more insight into the effects of peripheral humoral immune response involving these three molecules on underlying mechanisms of Parkinson’s disease. Moreover, it needs more comprehensive studies to validate the diagnostic and prognostic value of these three molecules in PD.

## Data availability statement

The original contributions presented in the study are included in the article/**Supplementary Materials**. Further inquiries can be directed to the corresponding author.

## Ethics statement

This study was reviewed and approved by Tianjin Huanhu Hospital. The patients/participants provided their written informed consent to participate in this study. Written informed consent was obtained from the individual(s) for the publication of any potentially identifiable images or data included in this article.

## Author contributions

Study design and data collection: NX and ZD. Data analysis and interpretation: PK and YH. Writing and review of the manuscript: NX, QW and XC. Review, supervision and project administration: BZ. All authors contributed to the article and approved the submitted version.

## Funding

This study was supported by a key project of the Tianjin Municipal Science and Technology Bureau, China (Grant No. 19JCZDJC35400).

## Acknowledgments

We gratefully acknowledge contributions from the Department of Clinical Laboratory of Tianjin Huanhu Hospital, and GEO databases.

## Conflict of interest

The authors declare that the research was conducted in the absence of any commercial or financial relationships that could be construed as a potential conflict of interest.

## Publisher’s note

All claims expressed in this article are solely those of the authors and do not necessarily represent those of their affiliated organizations, or those of the publisher, the editors and the reviewers. Any product that may be evaluated in this article, or claim that may be made by its manufacturer, is not guaranteed or endorsed by the publisher.

## References

[1] Balestrino R Schapira AHV. Parkinson Disease. Eur J Neurol (2019) 27(1):27–42. doi: 10.1111/ene.14108 31631455

[2] Bloem BR Okun MS Klein C. Parkinson’s disease. Lancet (2021) 397(10291):2284–303. doi: 10.1016/S0140-6736(21)00218-X 33848468

[3] Jankovic J Tan EK. Parkinson’s disease: Etiopathogenesis and treatment. J Neurol Neurosurg Psychiatry (2020) 91(8):795–808. doi: 10.1136/jnnp-2019-322338 32576618

[4] Marogianni C Sokratous M Dardiotis E Hadjigeorgiou GM Bogdanos D Xiromerisiou G. Neurodegeneration and inflammation–an interesting interplay in parkinson’s disease. Int J Mol Sci (2020) 21(22):8421. doi: 10.3390/ijms21228421 PMC7697354 33182554

[5] Tansey MG Wallings RL Houser MC Herrick MK Keating CE Joers V. Inflammation and immune dysfunction in Parkinson disease. Nat Rev Immunol (2022) 4:1–17. doi: 10.1038/s41577-022-00684-6 PMC8895080 35246670

[6] Sweeney MD Sagare AP Zlokovic BV. Blood–brain barrier breakdown in Alzheimer disease and other neurodegenerative disorders. Nat Rev Neurol (2018) 14(3):133–50. doi: 10.1038/nrneurol.2017.188 PMC5829048 29377008

[7] Galea I. The blood-brain barrier in systemic infection and inflammation. Cell Mol Immunol (2021) 18(11):2489–501. doi: 10.1038/s41423-021-00757-x PMC8481764 34594000

[8] Hickman S Izzy S Sen P Morsett L El Khoury J. Microglia in neurodegeneration. Nat Neurosci (2018) 21(10):1359–69. doi: 10.1038/s41593-018-0242-x PMC6817969 30258234

[9] Atik A Stewart T Zhang J. Alpha-synuclein as a biomarker for parkinson’s disease. Brain Pathol (2016) 26(3):410–8. doi: 10.1111/bpa.12370 PMC6245641 26940058

[10] Prinz M Priller J. The role of peripheral immune cells in the cns in steady state and disease. Nat Neurosci (2017) 20(2):136–44. doi: 10.1038/nn.4475 28092660

[11] Dekens DW Eisel ULM Gouweleeuw L Schoemaker RG De Deyn PP Naude PJW. Lipocalin 2 as a link between ageing, risk factor conditions and age-related brain diseases. Ageing Res Rev (2021) 70:101414. doi: 10.1016/j.arr.2021.101414 34325073

[12] Chen Z Chen S Liu J. The role of T cells in the pathogenesis of parkinson’s disease. Prog Neurobiol (2018) 169:1–23. doi: 10.1016/j.pneurobio.2018.08.002 30114440

[13] Munoz-Delgado L Macias-Garcia D Jesus S Martin-Rodriguez JF Labrador-Espinosa MA Jimenez-Jaraba MV. Peripheral immune profile and neutrophil-to-Lymphocyte ratio in parkinson’s disease. Mov Disord (2021) 36(10):2426–30. doi: 10.1002/mds.28685 34101890

[14] Harms AS Thome AD Yan Z Schonhoff AM Williams GP Li X. Peripheral monocyte entry is required for alpha-synuclein induced inflammation and neurodegeneration in a model of Parkinson disease. Exp Neurol (2018) 300:179–87. doi: 10.1016/j.expneurol.2017.11.010 PMC5759972 29155051

[15] Miki Y Shimoyama S Kon T Ueno T Hayakari R Tanji K. Alteration of autophagy-related proteins in peripheral blood mononuclear cells of patients with parkinson’s disease. Neurobiol Aging (2018) 63:33–43. doi: 10.1016/j.neurobiolaging.2017.11.006 29223072

[16] Mutez E Nkiliza A Belarbi K de Broucker A Vanbesien-Mailliot C Bleuse S. Involvement of the immune system, endocytosis and Eif2 signaling in both genetically determined and sporadic forms of parkinson’s disease. Neurobiol Dis (2014) 63:165–70. doi: 10.1016/j.nbd.2013.11.007 24269915

[17] Mutez E Larvor L Leprêtre F Mouroux V Hamalek D Kerckaert J-P. Transcriptional profile of Parkinson blood mononuclear cells with Lrrk2 mutation. Neurobiol Aging (2011) 32(10):1839–48. doi: 10.1016/j.neurobiolaging.2009.10.016 20096956

[18] Davis S Meltzer PS. Geoquery: A bridge between the gene expression omnibus (Geo) and bioconductor. Bioinformatics (2007) 23(14):1846–7. doi: 10.1093/bioinformatics/btm254 17496320

[19] Ritchie ME Phipson B Wu D Hu Y Law CW Shi W. Limma powers differential expression analyses for rna-sequencing and microarray studies. Nucleic Acids Res (2015) 43(7):e47–e. doi: 10.1093/nar/gkv007 PMC4402510 25605792

[20] Piñero J Bravo À Queralt-Rosinach N Gutiérrez-Sacristán A Deu-Pons J Centeno E. Disgenet: A comprehensive platform integrating information on human disease-associated genes and variants. Nucleic Acids Res (2017) 45(D1):D833–d9. doi: 10.1093/nar/gkw943 PMC5210640 27924018

[21] The Gene Ontology C. The gene ontology resource: 20 years and still going strong. Nucleic Acids Res (2019) 47(D1):D330–D8. doi: 10.1093/nar/gky1055 PMC6323945 30395331

[22] Kanehisa M Furumichi M Tanabe M Sato Y Morishima K. Kegg: New perspectives on genomes, pathways, diseases and drugs. Nucleic Acids Res (2017) 45(D1):D353–D61. doi: 10.1093/nar/gkw1092 PMC5210567 27899662

[23] Yu G Wang L-G Han Y He Q-Y. Clusterprofiler: An r package for comparing biological themes among gene clusters. OMICS: A J Integr Biol (2012) 16(5):284–7. doi: 10.1089/omi.2011.0118 PMC3339379 22455463

[24] Papa I Saliba D Ponzoni M Bustamante S Canete PF Gonzalez-Figueroa P. Tfh-derived dopamine accelerates productive synapses in germinal centres. Nature (2017) 547(7663):318–23. doi: 10.1038/nature23013 PMC5540173 28700579

[25] Toborek M Coley JS Calderon TM Gaskill PJ Eugenin EA Berman JW. Dopamine increases Cd14+Cd16+ monocyte migration and adhesion in the context of substance abuse and hiv neuropathogenesis. PloS One (2015) 10(2):e0117450. doi: 10.1371/journal.pone.0117450 25647501 PMC4315499

[26] Nissen SK Shrivastava K Schulte C Otzen DE Goldeck D Berg D. Alterations in blood monocyte functions in parkinson’s disease. Mov Disord (2019) 34(11):1711–21. doi: 10.1002/mds.27815 31449711

[27] Tan EK Chao YX West A Chan LL Poewe W Jankovic J. Parkinson Disease and the immune system - associations, mechanisms and therapeutics. Nat Rev Neurol (2020) 16(6):303–18. doi: 10.1038/s41582-020-0344-4 32332985

[28] Schonhoff AM Williams GP Wallen ZD Standaert DG Harms AS. Innate and adaptive immune responses in parkinson’s disease. Prog Brain Res (2020) 252:169–216. doi: 10.1016/bs.pbr.2019.10.006 32247364 PMC7185735

[29] Wang P Yao L Luo M Zhou W Jin X Xu Z. Single-cell transcriptome and tcr profiling reveal activated and expanded T cell populations in parkinson’s disease. Cell Discovery (2021) 7(1):52. doi: 10.1038/s41421-021-00280-3 34282123 PMC8289849

[30] Chen X Feng W Ou R Liu J Yang J Fu J. Evidence for peripheral immune activation in parkinson’s disease. Front Aging Neurosci (2021) 13:617370. doi: 10.3389/fnagi.2021.617370 33994989 PMC8119625

[31] Horvath I Iashchishyn IA Forsgren L Morozova-Roche LA. Immunochemical detection of alpha-synuclein autoantibodies in parkinson’s disease: Correlation between plasma and cerebrospinal fluid levels. ACS Chem Neurosci (2017) 8(6):1170–6. doi: 10.1021/acschemneuro.7b00063 28263550

[32] Folke J Bergholt E Pakkenberg B Aznar S Brudek T. Alpha-synuclein autoimmune decline in prodromal multiple system atrophy and parkinson’s disease. Int J Mol Sci (2022) 23(12):6554. doi: 10.3390/ijms23126554 35742998 PMC9224313

[33] Sun C Yu W Zhao Z Song C Liu Y Jia G. Peripheral humoral immune response is associated with the non-motor symptoms of parkinson’s disease. Front Neurosci (2019) 13:1057. doi: 10.3389/fnins.2019.01057 31649497 PMC6795918

[34] Hughes CE Nibbs RJB. A guide to chemokines and their receptors. FEBS J (2018) 285(16):2944–71. doi: 10.1111/febs.14466 PMC6120486 29637711

[35] Liu J-Q Chu S-F Zhou X Zhang D-Y Chen N-H. Role of chemokines in parkinson’s disease. Brain Res Bull (2019) 152:11–8. doi: 10.1016/j.brainresbull.2019.05.020 31136787

[36] Abdi IY Ghanem SS El-Agnaf OM. Immune-related biomarkers for parkinson’s disease. Neurobiol Dis (2022) 170:105771. doi: 10.1016/j.nbd.2022.105771 35598675

[37] Lau SF Fu AKY Ip NY. Cytokine signaling convergence regulates the microglial state transition in alzheimer’s disease. Cell Mol Life Sci (2021) 78(10):4703–12. doi: 10.1007/s00018-021-03810-0 PMC8195901 33847763

[38] Leighton SP Nerurkar L Krishnadas R Johnman C Graham GJ Cavanagh J. Chemokines in depression in health and in inflammatory illness: A systematic review and meta-analysis. Mol Psychiatry (2017) 23(1):48–58. doi: 10.1038/mp.2017.205 29133955 PMC5754468

[39] Li L Zhang L Zhang T Qi X Cheng G Xia L. Serum chemokine Cxcl7 as a potential novel biomarker for obstructive colorectal cancer. Front Oncol (2021) 10:599363. doi: 10.3389/fonc.2020.599363 33643903 PMC7902867

[40] Li L Zhang L Tian Y Zhang T Duan G Liu Y. Serum chemokine Cxcl7 as a diagnostic biomarker for colorectal cancer. Front Oncol (2019) 9:921. doi: 10.3389/fonc.2019.00921 31649870 PMC6794610

[41] Rajarathnam K Schnoor M Richardson RM Rajagopal S. How do chemokines navigate neutrophils to the target site: Dissecting the structural mechanisms and signaling pathways. Cell Signalling (2019) 54:69–80. doi: 10.1016/j.cellsig.2018.11.004 30465827 PMC6664297

[42] Choi Y-G Yeo S Hong Y-M Kim S-H Lim S. Changes of gene expression profiles in the cervical spinal cord by acupuncture in an mptp-intoxicated mouse model: Microarray analysis. Gene (2011) 481(1):7–16. doi: 10.1016/j.gene.2011.03.006 21440609

[43] Tondo G Perani D Comi C. Tam Receptor pathways at the crossroads of neuroinflammation and neurodegeneration. Dis Markers (2019) 2019:1–13. doi: 10.1155/2019/2387614 PMC6766163 31636733

[44] Hackeng TM van ‘t Veer C Meijers JC Bouma BN. Human protein s inhibits prothrombinase complex activity on endothelial cells and platelets *Via* direct interactions with factors va and xa. J Biol Chem (1994) 269(33):21051–8. doi: 10.1016/S0021-9258(17)31928-2 8063724

[45] Shafit-Zagardo B Gruber RC DuBois JC. The role of Tam family receptors and ligands in the nervous system: From development to pathobiology. Pharmacol Ther (2018) 188:97–117. doi: 10.1016/j.pharmthera.2018.03.002 29514053 PMC6067981

[46] Carrera Silva EA Chan PY Joannas L Errasti AE Gagliani N Bosurgi L. T Cell-derived protein s engages Tam receptor signaling in dendritic cells to control the magnitude of the immune response. Immunity (2013) 39(1):160–70. doi: 10.1016/j.immuni.2013.06.010 PMC4017237 23850380

[47] Ma GZM Giuffrida LL Gresle MM Haartsen J Laverick L Butzkueven H. Association of plasma levels of protein s with disease severity in multiple sclerosis. Multiple Sclerosis J - Experimental Trans Clin (2015) 1:2055217315596532. doi: 10.1177/2055217315596532 PMC5433335 28607700

[48] Kim DK Han D Park J Choi H Park J-C Cha M-Y. Deep proteome profiling of the hippocampus in the 5xfad mouse model reveals biological process alterations and a novel biomarker of alzheimer’s disease. Exp Mol Med (2019) 51(11):1–17. doi: 10.1038/s12276-019-0326-z PMC6856180 31727875

[49] Lim D Jeong JH Song J. Lipocalin 2 regulates iron homeostasis, neuroinflammation, and insulin resistance in the brains of patients with dementia: Evidence from the current literature. CNS Neurosci Ther (2021) 27(8):883–94. doi: 10.1111/cns.13653 PMC8265939 33945675

[50] Eidson LN Kannarkat GT Barnum CJ Chang J Chung J Caspell-Garcia C. Candidate inflammatory biomarkers display unique relationships with alpha-synuclein and correlate with measures of disease severity in subjects with parkinson’s disease. J Neuroinflamm (2017) 14(1):164. doi: 10.1186/s12974-017-0935-1 PMC5563061 28821274

[51] Kim BW Jeong KH Kim JH Jin M Kim JH Lee MG. Pathogenic upregulation of glial lipocalin-2 in the parkinsonian dopaminergic system. J Neurosci (2016) 36(20):5608–22. doi: 10.1523/jneurosci.4261-15.2016 PMC6601774 27194339

[52] Xiong M Qian Q Liang X Wei Y-D. Serum levels of lipocalin-2 in patients with parkinson’s disease. Neurol Sci (2021) 43(3):1755–9. doi: 10.1007/s10072-021-05579-3 34455500

[53] Eruysal E Ravdin L Kamel H Iadecola C Ishii M Zetterberg H. Plasma lipocalin-2 levels in the preclinical stage of alzheimer’s disease. Alzheimer’s Dementia: Diagnosis Assess Dis Monit (2019) 11(1):646–53. doi: 10.1016/j.dadm.2019.07.004 PMC6733778 31517027

[54] Llorens F Hermann P Villar-Piqué A Diaz-Lucena D Nägga K Hansson O. Cerebrospinal fluid lipocalin 2 as a novel biomarker for the differential diagnosis of vascular dementia. Nat Commun (2020) 11(1):619. doi: 10.1038/s41467-020-14373-2 32001681 PMC6992814

