# Supplementary material for: Identification and validation of key molecules associated with humoral immune modulation in Parkinson’s disease based on bioinformatics
Source: Front Immunol. 2022 Sep 15;13:948615. doi: 10.3389/fimmu.2022.948615 (PMC9520667; doi:10.3389/fimmu.2022.948615)
Supplement: Supplementary file 1 [file DataSheet_1.docx]

Supplemental Methods 1

A fasting sample of venous whole blood was drawn from each participant into the EDTA anticoagulant tube on the next day of hospital. RNA was extracted from the whole blood by Blood (liquid sample) total RNA rapid extraction kit (spin column type) (BioTeke, Beijing, China).

1. 0.75ml of lysis solution (RLS) was added to each 0.25ml of whole blood, and mixed several times by sample gun to help lyse the cells in the sample.

2. Vortex the homogenized sample vigorously for at least 1 minute, and incubated at 15-30°C for 5 minutes to decompose the ribosomes completely.

3. Optional: If there were obvious clumps of insoluble material in the solution, centrifuged at 12,000 rpm for 10 minutes at 4°C. Carefully removed the supernatant and transferred to a new RNase free centrifuge tube.

4. Add 0.2ml of chloroform and capped the centrifuge tube tightly, shaken vigorously for 15s, then incubated for 3 minutes at room temperature.

5. Centrifuge at 12000rpm at 4°C for 10 minutes, the sample would be divided into three layers; the lower organic phase, the middle layer and the upper colorless aqueous phase, and RNA existed in the aqueous phase. The volume of aqueous phase was about 60% of the above added lysis solution (RLS). Next the aqueous phase was transferred to a new tube.

6. Add equal volume of 70% absolute ethanol, inverted and mixed, then the solution and possible precipitates were transferred to the adsorption column (RA).

7. Centrifuge at 10000rpm for 45s, discarded the waste liquid, and put the adsorption column back into the collection tube.

8. Add 0.5ml of washing water (RW), centrifuged at 12000rpm for 60s, and discarded the waste solution. Put the adsorption column (RA) back into the empty collection tube, centrifuged at 12,000 rpm for 2 minutes, and tried to remove the washing water, so as to avoid the residual ethanol inhibiting the enzymatic digestion reaction.

9. Add 0.5ml of protein-removing solution (RE), placed at room temperature for 2 minutes, and centrifuged at 12000rpm for 45s, then discarded the waste solution.

10. Add 0.5ml of washing water (RW), centrifuged at 12000rpm for 60s, and discarded the waste solution.

11. Put the adsorption column (RA) back into the empty collection tube, centrifuged at 12,000 rpm for 2 minutes, and tried to remove the washing water (RW), so as to avoid the residual ethanol inhibiting the downstream reaction.

12. Take out the adsorption column (RA) and put it into an RNase free centrifuge tube. According to the expected RNA yield, add 30-50ul of RNase-free water to the middle of the adsorption membrane (pre-heating in a 65℃-70℃ water bath was better), placed at room temperature for 2 minutes, and centrifuged at 12,000rpm for 1 minute.

If more RNA was required, added the obtained solution to the centrifugal adsorption column (RA) and centrifuged for 1 minute, or added 30ul of RNase-free water once again and centrifuged for 1 minute, and combined the two eluates.
