# Supplementary figures and images for: Identification and validation of key molecules associated with humoral immune modulation in Parkinson’s disease based on bioinformatics
Source: Front Immunol. 2022 Sep 15;13:948615. doi: 10.3389/fimmu.2022.948615 (PMC9520667; doi:10.3389/fimmu.2022.948615)

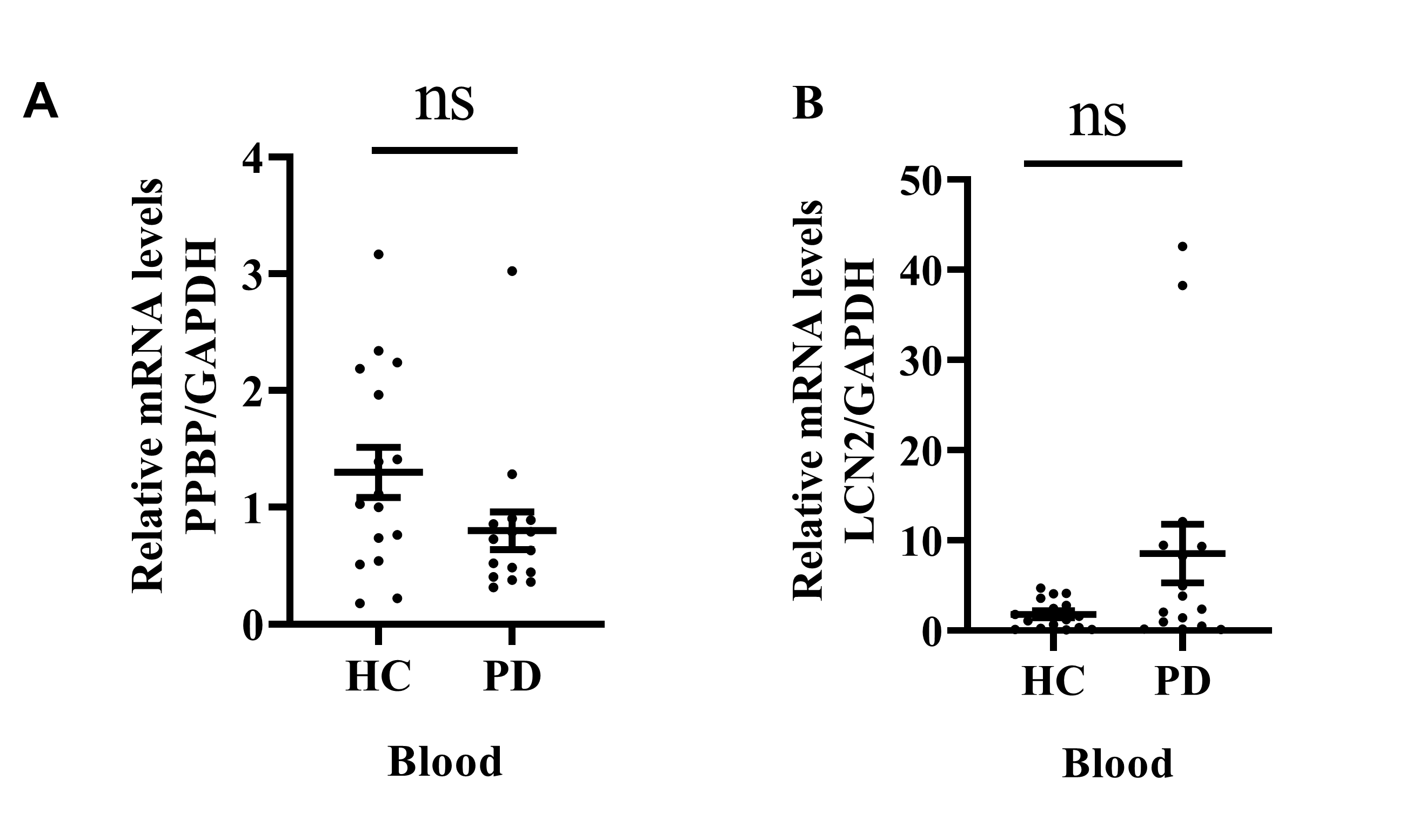

Supplement: Supplementary file 2 [file Image_1.tif]
