# Supplementary material for: Identification and validation of key molecules associated with humoral immune modulation in Parkinson’s disease based on bioinformatics
Source: Front Immunol. 2022 Sep 15;13:948615. doi: 10.3389/fimmu.2022.948615 (PMC9520667; doi:10.3389/fimmu.2022.948615)
Supplement: Supplementary file 3 [file Table_1.docx]

Supplemental Table 1 | the 94 differentially expressed PD-related genes

| Symbol | ENTREZID | Gene Name |
| --- | --- | --- |
| SEMA5A | 9037 | semaphorin 5A (SEMA5A) |
| CDA | 978 | cytidine deaminase (CDA) |
| IL1RN | 3557 | interleukin 1 receptor antagonist (IL1RN) |
| RNF11 | 26994 | ring finger protein 11 (RNF11) |
| HFE | 3077 | Hemochromatosis (HFE) |
| PROS1 | 5627 | protein S (alpha) (PROS1) |
| SERPINE1 | 5054 | serpin family E member 1 (SERPINE1) |
| HNMT | 3176 | histamine N-methyltransferase (HNMT) |
| CLU | 1191 | Clusterin (CLU) |
| SLC8A1 | 6546 | solute carrier family 8 member A1 (SLC8A1) |
| SLC6A4 | 6532 | solute carrier family 6 member 4 (SLC6A4) |
| IGF1R | 3480 | insulin like growth factor 1 receptor (IGF1R) |
| NRGN | 4900 | Neurogranin (NRGN) |
| ALDH2 | 217 | aldehyde dehydrogenase 2 family (mitochondrial) (ALDH2) |
| CA2 | 760 | carbonic anhydrase 2 (CA2) |
| CYP1B1 | 1545 | cytochrome P450 family 1 subfamily B member 1 (CYP1B1) |
| SIRPA | 140885 | signal regulatory protein alpha (SIRPA) |
| TIMP1 | 7076 | TIMP metallopeptidase inhibitor 1 (TIMP1) |
| CD36 | 948 | CD36 molecule (CD36) |
| CA8 | 767 | carbonic anhydrase 8 (CA8) |
| PGLYRP1 | 8993 | peptidoglycan recognition protein 1 (PGLYRP1) |
| CCR3 | 1232 | C-C motif chemokine receptor 3 (CCR3) |
| SYBU | 55638 | Syntabulin (SYBU) |
| CCR2 | 729230 | C-C motif chemokine receptor 2 (CCR2) |
| TGM2 | 7052 | transglutaminase 2 (TGM2) |
| GPX1 | 2876 | glutathione peroxidase 1 (GPX1) |
| DAPK1 | 1612 | death associated protein kinase 1 (DAPK1) |
| SLC11A1 | 6556 | solute carrier family 11 member 1 (SLC11A1) |
| TPM1 | 7168 | tropomyosin 1 (alpha) (TPM1) |
| ELOVL7 | 79993 | ELOVL fatty acid elongase 7 (ELOVL7) |
| RNASE1 | 6035 | ribonuclease A family member 1, pancreatic (RNASE1) |
| ARFGAP1 | 55738 | ADP ribosylation factor GTPase activating protein 1 (ARFGAP1) |
| DNM3 | 26052 | dynamin 3 (DNM3) |
| BACE2 | 25825 | beta-site APP-cleaving enzyme 2 (BACE2) |
| MS4A6A | 64231 | membrane spanning 4-domains A6A (MS4A6A) |
| MMRN1 | 22915 | multimerin 1 (MMRN1) |
| DNAJC6 | 9829 | DnaJ heat shock protein family (Hsp40) member C6 (DNAJC6) |
| TAL1 | 6886 | TAL bHLH transcription factor 1, erythroid differentiation factor (TAL1) |
| NCL | 4691 | Nucleolin (NCL) |
| IL1B | 3553 | interleukin 1 beta (IL1B) |
| ALDH1A1 | 216 | aldehyde dehydrogenase 1 family member A1 (ALDH1A1) |
| MALAT1 | 378938 | metastasis associated lung adenocarcinoma transcript 1 (non-protein coding) (MALAT1) |
| TLR4 | 7099 | toll like receptor 4 (TLR4) |
| PVALB | 5816 | Parvalbumin (PVALB) |
| CD44 | 960 | CD44 molecule (Indian blood group) (CD44) |
| MGLL | 11343 | monoglyceride lipase (MGLL) |
| CFB | 629 | complement factor B (CFB) |
| TLR2 | 7097 | toll like receptor 2 (TLR2) |
| GRN | 2896 | granulin precursor (GRN) |
| SLC45A3 | 85414 | solute carrier family 45 member 3 (SLC45A3) |
| RTN1 | 6252 | reticulon 1 (RTN1) |
| LRP1 | 4035 | LDL receptor related protein 1 (LRP1) |
| GSTT1 | 2952 | glutathione S-transferase theta 1 (GSTT1) |
| LYPD5 | 284348 | LY6/PLAUR domain containing 5 (LYPD5) |
| MEFV | 4210 | Mediterranean fever (MEFV) |
| ACACA | 31 | acetyl-CoA carboxylase alpha (ACACA) |
| EGFR | 1956 | epidermal growth factor receptor (EGFR) |
| CST3 | 1471 | cystatin C (CST3) |
| PPP3R1 | 5534 | protein phosphatase 3 regulatory subunit B, alpha (PPP3R1) |
| NT5E | 4907 | 5'-nucleotidase ecto (NT5E) |
| CNR1 | 1268 | cannabinoid receptor 1 (CNR1) |
| NLRP3 | 114548 | NLR family pyrin domain containing 3 (NLRP3) |
| CD14 | 929 | CD14 molecule (CD14) |
| PCNT | 5116 | Pericentrin (PCNT) |
| ANKRD50 | 57182 | ankyrin repeat domain 50 (ANKRD50) |
| SNCA | 6622 | synuclein alpha (SNCA) |
| ABCA1 | 19 | ATP binding cassette subfamily A member 1 (ABCA1) |
| NFE2 | 4778 | nuclear factor, erythroid 2 (NFE2) |
| IL33 | 90865 | interleukin 33 (IL33) |
| TGFB2 | 7042 | transforming growth factor beta 2 (TGFB2) |
| ANGPT1 | 284 | angiopoietin 1 (ANGPT1) |
| VDR | 7421 | vitamin D (1,25-dihydroxyvitamin D3) receptor (VDR) |
| KCNIP4 | 80333 | potassium voltage-gated channel interacting protein 4 (KCNIP4) |
| IGF2 | 3481 | insulin like growth factor 2 (IGF2) |
| PPBP | 5473 | pro-platelet basic protein (PPBP) |
| SOD2 | 6648 | superoxide dismutase 2, mitochondrial (SOD2) |
| VEGFA | 7422 | vascular endothelial growth factor A (VEGFA) |
| SLC2A9 | 56606 | solute carrier family 2 member 9 (SLC2A9) |
| NR4A2 | 4929 | nuclear receptor subfamily 4 group A member 2 (NR4A2) |
| FKBP1A | 2280 | FK506 binding protein 1A (FKBP1A) |
| IL4 | 3565 | interleukin 4 (IL4) |
| BST1 | 683 | bone marrow stromal cell antigen 1 (BST1) |
| GJB2 | 2706 | gap junction protein beta 2 (GJB2) |
| P2RX5 | 5026 | purinergic receptor P2X 5 (P2RX5) |
| MIR223 | 407008 | microRNA 223 (MIR223) |
| TEF | 7008 | TEF, PAR bZIP transcription factor (TEF) |
| SCD | 6319 | stearoyl-CoA desaturase (SCD) |
| DLG4 | 1742 | discs large MAGUK scaffold protein 4 (DLG4) |
| P2RX1 | 5023 | purinergic receptor P2X 1 (P2RX1) |
| LCN2 | 3934 | lipocalin 2 (LCN2) |
| RGS10 | 6001 | regulator of G-protein signaling 10 (RGS10) |
| CD24 | 100133941 | CD24 molecule (CD24) |
| GLA | 2717 | galactosidase alpha (GLA) |
| CD200 | 4345 | CD200 molecule (CD200) |
