# Supplementary material for: Identification and validation of key molecules associated with humoral immune modulation in Parkinson’s disease based on bioinformatics
Source: Front Immunol. 2022 Sep 15;13:948615. doi: 10.3389/fimmu.2022.948615 (PMC9520667; doi:10.3389/fimmu.2022.948615)
Supplement: Supplementary file 4 [file Table_2.docx]

Supplemental Table 2 | the immune-related pathways in the term of biological processes.

| ID | Description | Pvalue | Symbol | Count |
| --- | --- | --- | --- | --- |
| GO:0002697 | regulation of immune effector process | 0.0000 | PROS1/CLU/NLRP3/CCR2/IL33/TGFB2/IL4/IL1B/TLR4 | 9 |
| GO:0002440 | production of molecular mediator of immune response | 0.0000 | NLRP3/CCR2/IL33/TGFB2/IL4/IL1B/TLR4/TLR2 | 8 |
| GO:0002367 | cytokine production involved in immune response | 0.0000 | NLRP3/CCR2/TGFB2/IL4/IL1B/TLR4/TLR2 | 7 |
| GO:0002700 | regulation of production of molecular mediator of immune response | 0.0000 | NLRP3/CCR2/IL33/TGFB2/IL4/IL1B/TLR4 | 7 |
| GO:0002460 | adaptive immune response based on somatic recombination of immune receptors built from immunoglobulin superfamily domains | 0.0000 | CLU/NLRP3/CCR2/IL33/IL4/IL1B/TLR4 | 7 |
| GO:0002699 | positive regulation of immune effector process | 0.0000 | NLRP3/CCR2/IL33/IL4/IL1B/TLR4 | 6 |
| GO:0006959 | humoral immune response | 0.0000 | PROS1/CLU/CCR2/PPBP/IL1B/LCN2 | 6 |
| GO:0002718 | regulation of cytokine production involved in immune response | 0.0000 | NLRP3/CCR2/TGFB2/IL1B/TLR4 | 5 |
| GO:0002702 | positive regulation of production of molecular mediator of immune response | 0.0000 | NLRP3/IL33/IL4/IL1B/TLR4 | 5 |
| GO:0002822 | regulation of adaptive immune response based on somatic recombination of immune receptors built from immunoglobulin superfamily domains | 0.0000 | NLRP3/CCR2/IL33/IL4/IL1B | 5 |
|  |  |  |  |  |
| GO:0002819 | regulation of adaptive immune response | 0.0000 | NLRP3/CCR2/IL33/IL4/IL1B | 5 |
| GO:0002683 | negative regulation of immune system process | 0.0004 | CCR2/IL33/TGFB2/IL4/TLR4 | 5 |
| GO:0042092 | type 2 immune response | 0.0000 | NLRP3/CCR2/IL33/IL4 | 4 |
| GO:0042088 | T-helper 1 type immune response | 0.0000 | CCR2/IL33/IL1B/TLR4 | 4 |
| GO:0002824 | positive regulation of adaptive immune response based on somatic recombination of immune receptors built from immunoglobulin superfamily domains | 0.0000 | NLRP3/CCR2/IL4/IL1B | 4 |
| GO:0002821 | positive regulation of adaptive immune response | 0.0000 | NLRP3/CCR2/IL4/IL1B | 4 |
| GO:0002698 | negative regulation of immune effector process | 0.0000 | CCR2/IL33/TGFB2/IL4 | 4 |
| GO:0050777 | negative regulation of immune response | 0.0001 | CCR2/IL33/TGFB2/IL4 | 4 |
| GO:0002283 | neutrophil activation involved in immune response | 0.0049 | PPBP/LCN2/CD44/TLR2 | 4 |
| GO:0002825 | regulation of T-helper 1 type immune response | 0.0000 | CCR2/IL33/IL1B | 3 |
| GO:0002828 | regulation of type 2 immune response | 0.0000 | NLRP3/CCR2/IL33 | 3 |
| GO:0002720 | positive regulation of cytokine production involved in immune response | 0.0001 | NLRP3/IL1B/TLR4 | 3 |
| GO:0002920 | regulation of humoral immune response | 0.0009 | PROS1/CLU/IL1B | 3 |
| GO:0002285 | lymphocyte activation involved in immune response | 0.0024 | NLRP3/IL4/TLR4 | 3 |
| GO:0002830 | positive regulation of type 2 immune response | 0.0002 | NLRP3/IL33 | 2 |
| GO:0002827 | positive regulation of T-helper 1 type immune response | 0.0004 | CCR2/IL1B | 2 |
| GO:2000316 | regulation of T-helper 17 type immune response | 0.0004 | NLRP3/IL4 | 2 |
| GO:0072538 | T-helper 17 type immune response | 0.0009 | NLRP3/IL4 | 2 |
| GO:0002701 | negative regulation of production of molecular mediator of immune response | 0.0012 | IL33/TGFB2 | 2 |
| GO:0002823 | negative regulation of adaptive immune response based on somatic recombination of immune receptors built from immunoglobulin superfamily domains | 0.0017 | IL33/IL4 | 2 |
| GO:0002820 | negative regulation of adaptive immune response | 0.0021 | IL33/IL4 | 2 |
| GO:0002294 | CD4-positive, alpha-beta T cell differentiation involved in immune response | 0.0035 | NLRP3/IL4 | 2 |
| GO:0002287 | alpha-beta T cell activation involved in immune response | 0.0037 | NLRP3/IL4 | 2 |
| GO:0002293 | alpha-beta T cell differentiation involved in immune response | 0.0037 | NLRP3/IL4 | 2 |
| GO:0002292 | T cell differentiation involved in immune response | 0.0046 | NLRP3/IL4 | 2 |
| GO:0002312 | B cell activation involved in immune response | 0.0058 | IL4/TLR4 | 2 |
| GO:0002286 | T cell activation involved in immune response | 0.0106 | NLRP3/IL4 | 2 |
| GO:2000317 | negative regulation of T-helper 17 type immune response | 0.0156 | IL4 | 1 |
| GO:2000318 | positive regulation of T-helper 17 type immune response | 0.0156 | NLRP3 | 1 |
